# Development of Nanoporous AAO Membrane for Nano Filtration Using the Acoustophoresis Method

**DOI:** 10.3390/s20143833

**Published:** 2020-07-09

**Authors:** Yatinkumar Patel, Giedrius Janusas, Arvydas Palevicius, Andrius Vilkauskas

**Affiliations:** Faculty of Mechanical Engineering and Design, Kaunas University of Technology, Studentu str. 56, LT-51424 Kaunas, Lithuania; giedrius.janusas@ktu.lt (G.J.); arvydas.palevicius@ktu.lt (A.P.); andrius.vilkauskas@ktu.lt (A.V.)

**Keywords:** nanoporous aluminum oxide, two-step anodization, nano filtration, contact angle, surface acoustic waves, acoustophoresis, micro-hydro mechanical system

## Abstract

A concept of a nanoporous anodic aluminum oxide (AAO) membrane as a vibro-active micro/nano-filter in a micro hydro mechanical system for the filtration, separation, and manipulation of bioparticles is reported in this paper. For the fabrication of a nanoporous AAO, a two-step mild anodization (MA) and hard anodization (HA) technique was used. Atomic force microscopy (AFM) and scanning electron microscopy (SEM) were used to analyze the surface morphology of nanoporous AAO. A nanoporous structure with a pore diameter in the range of 50–90 nm, an interpore distance of 110 nm, and an oxide layer thickness of 0.12 mm with 60.72% porosity was obtained. Fourier-transform infrared spectroscopy (FTIR) and energy-dispersive X-ray spectroscopy (EDS) were employed to evaluate AAO chemical properties. The obtained results showed that the AAO structure is of hexagonal symmetry and showed where Al_2_O_3_ is dominant. The hydrophobic properties of the nanoporous surface were characterized by water contact angle measurement. It was observed that the surface of the nanoporous AAO membrane is hydrophilic. Furthermore, to determine whether a nanomembrane could function as a vibro-active nano filter, a numerical simulation was performed using COMSOL Multiphysics 5.4 (COMSOL Inc, Stockholm, Sweden). Here, a membrane was excited at a frequency range of 0–100 kHz for surface acoustics wave (SAW) distribution on the surface of the nanoporous AAO using a PZT 5H cylinder (Piezo Hannas, Wuhan, China). The SAW, standing acoustic waves, and travelling acoustic waves of different wavelengths were excited to the fabricated AAO membrane and the results were compared with experimental ones, obtained from non-destructive testing method 3D scanning vibrometer (PSV-500-3D-HV, Polytec GmbH, Waldbronn, Germany) and holographic interferometry system (PRISM, Hy-Tech Forming Systems (USA), Phoenix, AZ, USA). Finally, a simulation of a single nanotube was performed to analyze the acoustic pressure distribution and time, needed to center nanoparticles in the nanotube.

## 1. Introduction

In recent years, scientists are endeavoring to develop a relatively efficacious and facile method for the formation of nanostructures such as nanopores, nanotubes, nanorods, and nanowires used in genuine application fields due to their excellent physical, chemical, mechanical, and optical properties [1,2,3,4,5,6]. In the development of microstructures, it is worth to have the outside dimensions to facilely control the internal structure. There have been a variety of innovations and practical application in the field of biomedical research [7,8]. A wide range of materials such as metals, ceramics, polymeric, and nanoporous oxides such as alumina, titania, zirconia, and silica have previously been developed for nano structure fabrication [9]. Many different synthesis techniques exist to fabricate well-ordered nanoporous structures such as lithography [10], X-ray [11], ion-beam [12], atomic layer deposition [13], micromachining [14], powder sintering [15], sol-gel [16], phase separation, and ion track etching [17,18]. However, those techniques require expensive and high-tech setup of equipment and laboratory work.

Among all the prominent methods for fabrication there is one called electro chemical anodization, where a nanoporous aluminum oxide membrane is used in the process [19,20]. Anodic aluminum oxide (AAO) attracts a great amount of attention due to its regular pore structure array, simplicity of control of the pore diameter across the surface area, low fabrication cost, high surface area, phenomenal thermal conductivity, non-destructiveness, and biocompatibility [21]. Because of these qualities, anodic alumina is broadly utilized in a broad range of applications, such as filtration processes [22], drug delivery [23], biosensing [24], oxygen sensing [25], corrosion resistors [26], catalysts [27], photograph catalysts [28], DNA sensors [29], cancer treatments [30], nanoparticle separation [31], electrochemical biosensors [32], and fluorescence detectors [33]. Besides, geometrical nanopore empowers researchers to utilize alumina as a template for the synthesis of nanostructures [34,35]. In a number of applications, controlling the nanostructure plays a pivotal role; however, in the case of anodic alumina, it can be easily controlled by customizing the electrochemical process parameters such as the duration of the process, the electrolytes used for anodization, and the method of anodization [36]. For a two-step anodization process (mild anodization and hard anodization) [19], in the first step, oxide layers with non-structured and irregular pores are formed [2,4,5,19] which can be removed by chemical etching, and in the second step, the formation of regular pores in the hexagonal array occurs [19,37].

By applying surface acoustic waves (SAW), it is possible to control the geometry of the internal structure in micro fluidics. SAW devices have been widely used in particle concentrations and bioparticle suspensions because of their predominant merits including small scale, wide frequency range, and good stability. Many methods for the manipulation of drops have been noted before, such as dielectrophoretic force [38], travelling wave dielectrophoretic [39], and electrowetting-on-dielectric [40]. Here, SAW is excited on a lithium niobite substrate by applying the AC signal on an actuating element on the surface of the substrate [41,42,43,44]. Additionally, it is conceivable to utilize the produced standing and travelling waves that excite the internal geometry of the fabricated microstructure, such as microchannels and nanotubes [45]. The progression of the microfluid can be seen by the vibration modes in the walls of the micro/nano hydrodynamic system [46]. Combing the PZT (piezoelectric) component ensures increases the viability and usefulness of the micro/nano dynamical system [47]. SAW is extensively used for a variety of microscale operations in flow actuation and in microfluidic devices [45], although this has largely been confined to micron dimension particles to date [45,48]. Also, the acoustophoresis manipulation of bioparticle suspension and separation using an AAO nanoporous membrane integrated with piezoelectric transducer by employing high frequency can make it easier to manipulate the nanoparticles in microfluidics and nanodevices for nanofiltration. An analysis of the existing and future developments of self-ordered nanoporous AAO membrane development systems [49] clearly shows that the formulated research problem in this paper is relevant and requires further attention.

**Figure 1:**
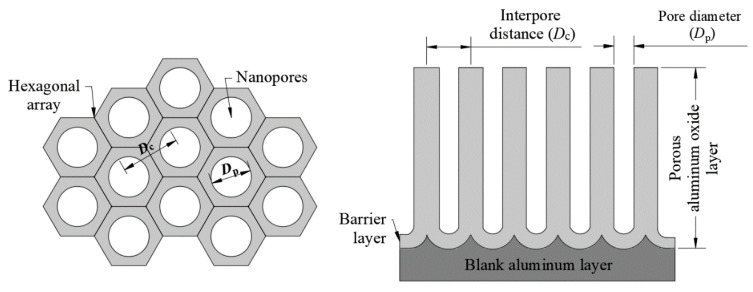
Schematic view of the nanoporous aluminum oxide template hexagonallyarranged structure.

In this research paper, a fabrication process of nanoporous anodic aluminum oxide (AAO) membrane is presented. A schematic view of a typical hexagonally arranged nanoporous AAO, which can be used as a vibro-active nano filter in a biomedical micro hydraulic device, is given in Figure 1.

The main aim of this research paper is to fabricate a nanoporous AAO membrane. The designed membrane can be used as a vibro-active nano filter for the filtration, separation, and transportation of particles, using standing surface acoustic waves (SSAW) to control the internal geometry of the nanotubes. First, a two-step anodization process for the fabrication of the nanoporous AAO membrane was undertaken. Its surface morphology was analyzed using atomic force microscopy (AFM) and scanning electron microscopy (SEM); its chemical elemental analysis was done by Fourier-transform infrared spectroscopy (FTIR) and energy dispersive X-Ray spectroscopy (EDS). Next, a hydrophobicity analysis of the membrane surface was carried out using contact angle measurements. Finally, a simulation was carried out to examine the behavior of the periodically actuated membrane using a PZT cylinder, by COMSOL Multiphysics 5.4 (COMSOL Inc, Stockholm, Sweden). The theoretical (simulation) results were compared with the experimental results, which were obtained using a non-destructive testing method—a 3D scanning vibrometer PSV-500-3D-HV (Polytec GmbH, Waldbronn, Germany) and holographic interferometry (PRISM, Hy-Tech Forming Systems (USA), Phoenix, AZ, USA). The results confirmed that standing acoustic waves are acting on the surface of the designed membrane.

## 2. Materials and Fabrication

### Fabrication of Nanoporous AAO Membrane

**Figure 2:**
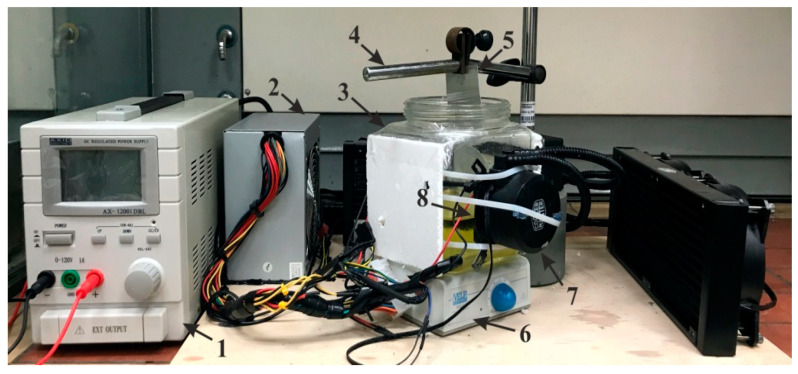
Experimental setup for the fabrication of a nanoporous anodic aluminum oxide (AAO) membrane: 1, a DC power unit; 2, a power supply unit for the Peltier element and master cooler; 3, a glass jar for electrolyte bath; 4, a holder for specimen; 5, A specimen (aluminum sheet); 6, a magnetic stirrer; 7, a master cooler (MLW-D24M, Cooler Master Technology Inc, Taipei, Taiwan) for the Peltier element; and 8, the Peltier element (TEC112715, Hebei I.T. (Shanghai) Co., Ltd., Shanghai, China).

**Figure 3:**
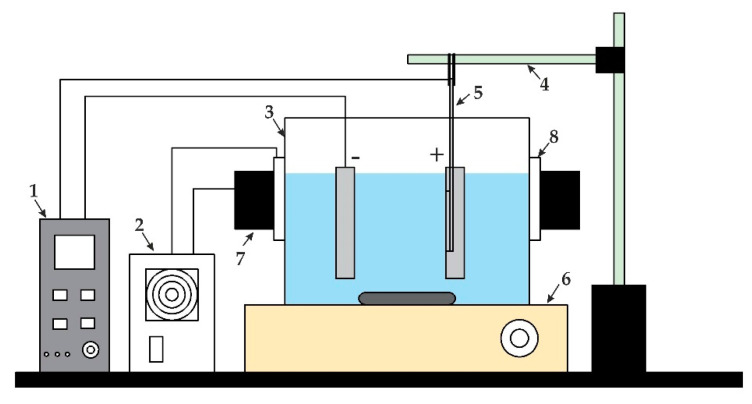
Schematic diagram of electrochemical anodization: 1, a DC power unit; 2, a power supply unit for the Peltier element and master cooler; 3, a glass jar for electrolyte bath; 4, a holder for specimen; 5, A specimen (aluminum sheet); 6, a magnetic stirrer; 7, a master cooler (MLW-D24M) for the Peltier element; and 8, the Peltier element (TEC112715).

A natural aluminum sheet of 0.3 mm thickness was used as the raw material for the fabrication of the nanoporous anodic aluminum oxide (AAO) membrane. The electrochemical anodization method (including two-step mild anodization (MA) and hard anodization (HA)) was applied for the fabrication of the AAO membrane. A custom-made experimental setup for two-step anodization is shown in Figure 2 and a schematic diagram of the experimental setup is presented in Figure 3.

**Figure 4:**
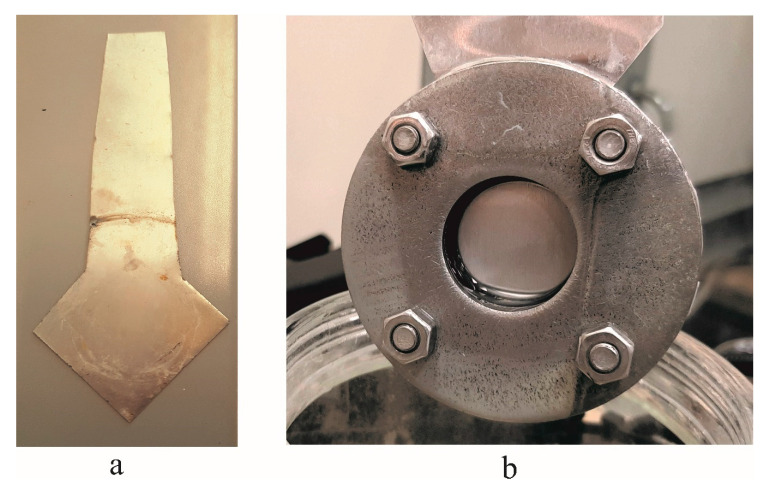
Specimen before fixing inside the holder (**a**), and custom-made specimen holder (**b**).

Before beginning the experimental setup (Figure 2) of the electrochemical anodization [12], a large aluminum sheet was cut into a square-shaped sample 10 × 10 cm in size and ultrasonically cleaned with acetone (C_3_H_6_O) for 5 min. The mechanical cleaning was done with the buffing disc until the surface of the aluminum sheet shone like a mirror and then cleaned with acetone (C_3_H_6_O). The cleaned sheet was then was cut into a specific shape (Figure 4a) to be easily fixed in the specimen holder (open area 30 mm diameter) (Figure 4b). Later, the sample was immersed in concentrated phosphoric acid (H_3_PO_4_) for 5 min in order to make surface hydrophilic without losing the gloss of the aluminum surface (during this step, the surface of the aluminum was covered with dense hydrogen bubbles).

Before anodization to achieve a low surface roughness of the sample, an electro-chemical polishing was performed at 20 V for 1 min with a solution of phosphoric acid (H_3_PO_4_), sulfuric acid (H_2_SO_4_), and water in a proportion of 2 : 2 : 1 (by volume), respectively, at a room temperature.

**Figure 5:**
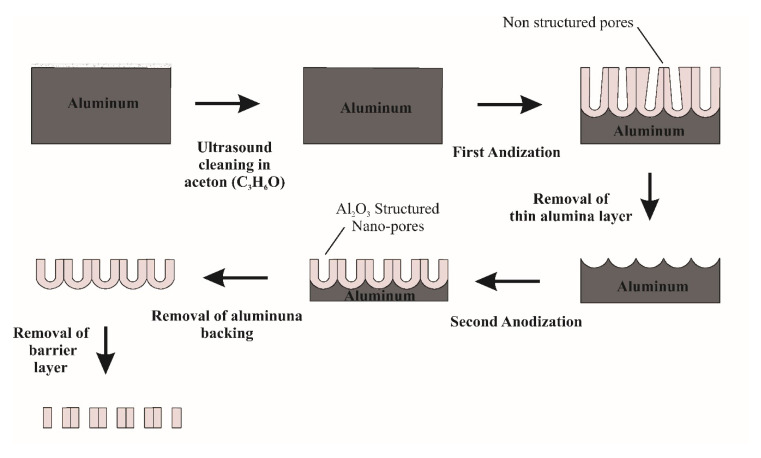
Scheme of the two-step anodization process for the fabrication of a nanoporous aluminum oxide membrane.

After surface preparation, the aluminum sheet was subjected to a two-step anodization process. A schematic diagram of the two-step anodization process for the fabrication of a nanoporous aluminum oxide membrane is given in Figure 5.

First, a mild anodization (MA) process was done: a potentiostatic regime at an identical voltage of 60 V was used for 1 h in an aqueous solution (1 L distilled water) of oxalic acid (C_2_H_2_O_4_) 0.3 M at a constant temperature of 5–8 °C, and maintained during the electrolyte bath by two Peltier elements, attached on jar; then a magnetic stirrer was placed under the electrolyte bath jar to continuously dissolve the solution. After the MA process, the formed layer of disordered aluminum oxide was removed by the chemical etching process using a mixture of a 3.5% concentrated phosphoric acid (H_3_PO_4_) and 2% chromium anhydride (CrO_3_) acid solution (by volume) in water (for 1 h at 50 °C). After etching, the specimen and the specimen holder were rinsed with distilled water for the second anodization step. In the second step, a hard anodization (HA) process was done with a duration time of 8 h in aqueous solution (1 L distilled water) of oxalic acid (C_2_H_2_O_4_) 0.3 M at a constant temperature of 5–8 °C. After HA, the specimen was removed from the holder and rinsed with water, then flipped and fixed in the holder for the etching of the bottom layer. The etching of the bottom layer was done using a solution of concentrated hydrochloric acid (HCl, 50%) with distilled water 50% (1 : 1 by volume). The bottom layer was etched for 3 min in solution and then rinsed with distilled water. The closed ends of the pores were opened by immersing the specimen in a solution of 3.5% concentrated phosphoric acid (H_3_PO_4_), 2% chromic acid (CrO3), and 94.5% distilled water for 10 min. According to the literature, the distance between the pores and the diameter depends on the electrolyte used for the anodization; in our work, a 40–100 nm diameter with the inter pore distance of 80–200 nm was achieved by using oxalic acid [50]. Thus, our results comply with the experimental results in other research works.

## 3. Experimental Results of Nanoporous AAO Membrane

### 3.1. Surface Morphology of Nanoporous AAO Membrane

**Figure 6:**
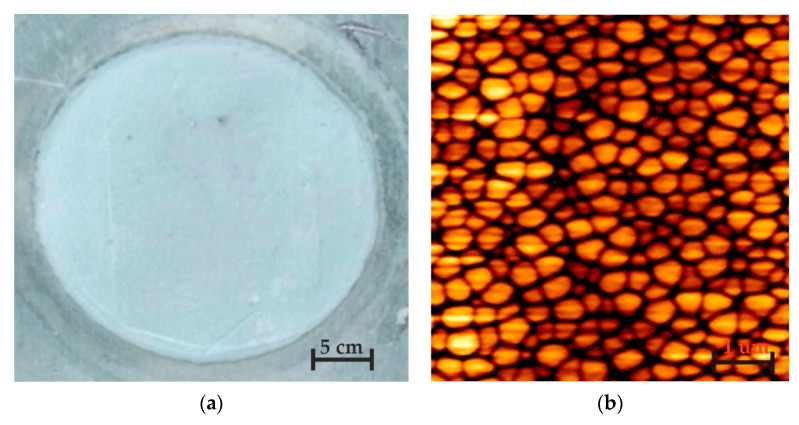
A photo of AAO, and (**a**) surface morphology of the (**b**) nanoporous aluminum oxide membrane.

For the surface morphology and topography measurements, atomic force microscope (AFM, Veeco/TM CP-RII SPM system, SPECS GmbH, Berlin, Germany) was used. AFM has multiple operating modes, with a scanning field of 100 × 100 µm, a height up to 15 µm with an area for samples that is up to 100 mm in diameter and 50 mm in height. A photo of a fabricated nanoporous AAO membrane using two-step anodization (oxalic acid) is presented in Figure 6a. The surface morphology of the produced element with a 2D view of arbitrary selections sized 3 × 3 µm is given in Figure 6b.

According to the literature [50], a hexagonal structure should be incurred during the anodization process. Thus, the experimental results show the distribution of nanopores on the surface of nanomembrane with a pore diameter of 50–90 nm. The interpore distance is more uniform (i.e., variation does not exceed 10%). In accordance with the literature [50], 40–100 nm pore diameter and 80–200 nm interpore distance were formed using oxalic acid as the electrolyte at 30–80 V DC for anodization. The porosity of the samples was estimated assuming an ideal hexagonal arrangement of pores in the structure using Equation (1), below:

(1) $$P=\frac{\pi }{2\sqrt{3}}{\left(\frac{D_{p}}{D_{c}}\right)}^{2}$$

where *D*_p_ and *D*_c_ are the pore diameter and the interpore distance, respectively. The density of pores, defined as the total number of pores occupying a surface area of 1 cm^2^, can be expressed by the following Equation (2):

(2) $$n=\frac{10^{14}}{\sqrt{3}*D_{c}{}^{2}}$$

Since the interpore distance is dependent on the anodization potential, a decrease in the pore density with the increasing anodization potential should be observed. The calculation results are given in Table 1.

**Figure 7:**
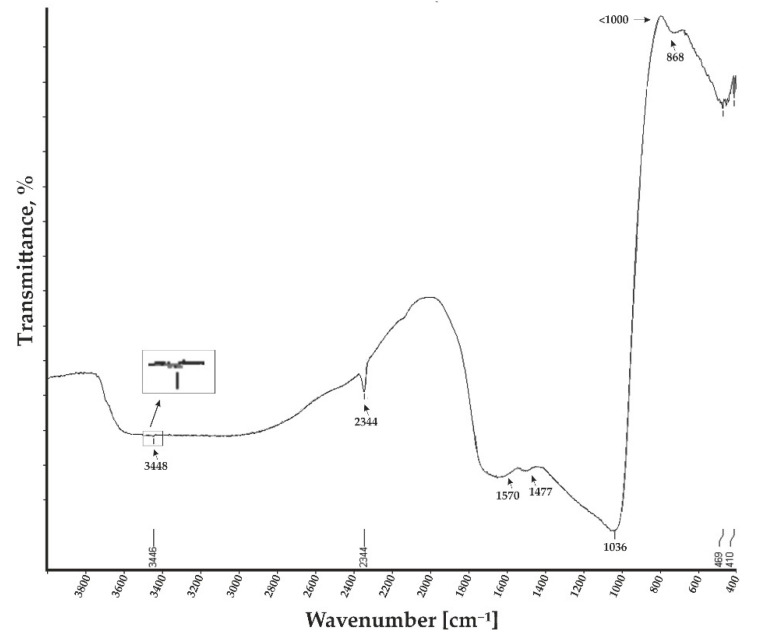
Fourier-transform infrared (FTIR) spectra of the anodized nanoporous aluminum oxide membrane for a wavenumber of 4000–400 cm^−1^.

Further, a FTIR analysis of the nanoporous aluminum oxide membrane was done. The FTIR transmittance spectrum at 4000–400 cm^−1^ of the Al_2_O_3_ membrane is presented in Figure 7.

In the FTIR spectra, strong and weak peaks were observed at 3448 cm^−1^, 2344 cm^−1^, 1570 cm^−1^, 1477 cm^−1^, 1036 cm^−1^, and <1000 cm^−1^. The stretching of the O-H bonds was visible at 3448 cm^−1^ due to the presence of the water molecules in the structure. The small vibrational peak was observed at 2344 cm^−1^ due to the link of C-O bond. A small symmetric and asymmetric stretching peak was observed between 1570 cm^−1^ and 1477 cm^−1^ due to carboxyl anions. The major peak around 1036–1000 cm^−1^ is characteristic of the complex vibration of the Al-O bond. The entire cluster of peaks compared to the theoretical and literature FTIR transmittance spectra of Al_2_O_3_ [51,52] reveals that nanoporous aluminum oxide membranes were formed.

### 3.2. SEM Based EDS Analysis of Fabricated Aluminum Oxide Membrane

**Figure 8:**
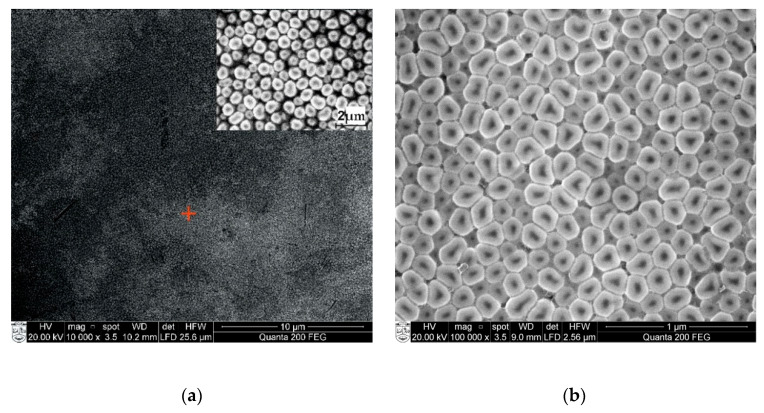
Scanning electron microscopy (SEM) top views of (**a**) nanoporous structures together with higher magnification (right-hand side insets 2 µm) and (**b**) the developed hexagonal honeycomb structure of the nanopore array (marker place in (**a**)) after the two-step step anodization in 0.3 M oxalic acid at 60 V.

The surface morphology and synthetic structure of the manufactured nanoporous alumina were assessed with scanning electron microscopy (SEM) FEI Quanta 200 FEG (FEI, Hillsboro, OR, USA), which includes the energy dispersive X-ray spectrometer (EDS) (a finder XFlash 4030, Bruker AXS GmbH Microanalysis, Karlsruhe, Germany). The AAO was examined under a controlled water steam environment. The maximal reachable limit for high-vacuum (<6 × 10^−4^ Pa) was 1.2 nm, for low-vacuum (10 to 130 Pa), it was 2.5 nm, and for extreme vacuum mode (10 to 4000 Pa), it was 3 nm. The EDS identifier permits the recognition of components ranging from boron (Z = 5) to americium (Z = 95). The chemical investigation can be resolved at the picked point, along the line or distribution on a surface. A modern 30 mm^2^ region solid state float identifier is cooled with Peltier component and gives 133 eV (at Mn K) energy resolution at 100,000 cps. Also, X-ray spectroscopy technique allows for the dissection of energy dispersions, for example, the energy contrasts between the different quantum conditions of a system and the probabilities that the system bounces between these states. SEM images of nanoporous AAO membrane, fabricated at DC 60 V using a two-step anodization method in a 0.3 M oxalic acid (C_2_H_2_O_4_) aqueous solution, are shown in Figure 8.

**Figure 9:**
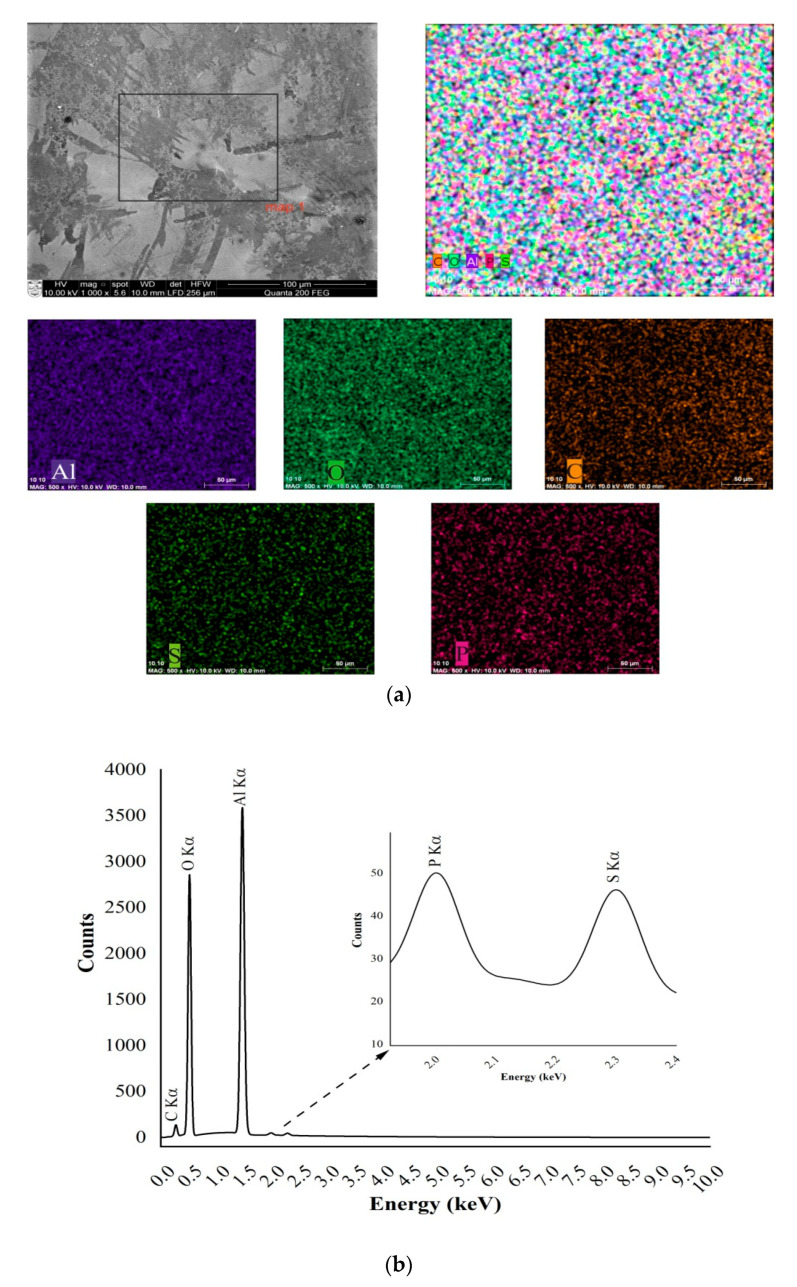
(**a**) SEM image together with split channel shows the presence of aluminum (Al), oxygen (O), carbon (C), phosphorus (P), and sulphur (S) on the surface of the fabricated AAO nanoporous membrane; and (**b**) energy dispersive spectrum of the nanoporous membrane.

The SEM image and EDS spectrum of the fabricated nanoporous AAO membrane are given in Figure 9. Figure 9a shows an examined area with a red marker (inset of 2 µm magnification) and Figure 8b shows a higher magnification of the examined area of the membrane. The images reveal that the honeycomb structures (hexagonal) were formed with a pore diameter between 50–90 nm and a 110 ± 10 interpore distance. Also, it is possible to control the geometry of the pore and interpore distance by regulating the different voltage and electrolyte ranges. The full chemical composition and elemental analysis made of an oxide based fabricated nanoporous AAO membrane can be seen in Figure 9.

Energy dispersive X-ray spectroscopy (EDS) analysis gives qualitative and quantitative determinations of the elemental composition of the formed nanoporous AAO membrane. The full composition is presented in Figure 9b. These microanalyses show the dominance of Al_2_O_3_ (aluminum oxide), aluminum with a Kα peak at 1.479 keV, and oxygen with a Kα peak at 0.5 keV (Figure 9b). Other peaks show smaller quantities of carbon (Kα peak at 0.37 keV), phosphorous (Kα peak at 2.01 keV), and sulphur (Kα peak at 2.307 keV). More detailed information on the normalized concentrations of the weight and the atomic percentages of the chemical composition of formed nanoporous AAO membranes is given in Table 2.

### 3.3. Hydrophobicity Analysis of Nanoporous AAO Membrane

The fabricated nanoporous AAO contains formed nano tubes throughout the entire surface of the membrane. Since this membrane is intended to use for nano filtration, the hydrophobic property of the formed nanoporous surface plays a crucial role. The hydrophobicity was evaluated by measuring the contact angle interface between the drop of the fluid and the AAO surface. A testing protocol has been designed and developed for the direct measurement of the contact angle on the surface of a nanoporous AAO membrane with a high purity probe. Three type liquids were used—distilled water, glycerin, and spirit.

**Figure 10:**
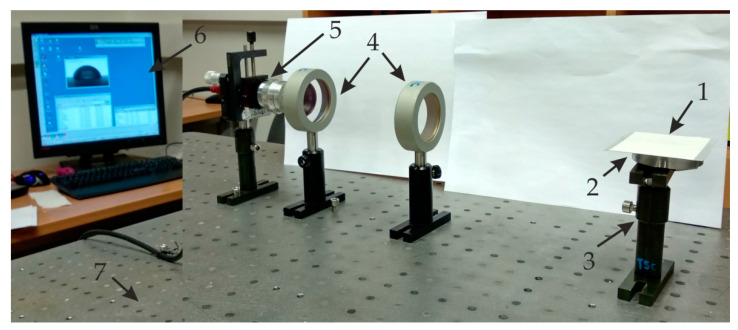
Experimental setup for the hydrophobic analysis of the AAO membrane contact angle measurements: 1, drop on the specimen; 2, the analyzed AAO specimen; 3, adjustable stand for specimen; 4, double convex lenses; 5, Guppy F-503 B&W CMOS camera; 6, computer system for analysis of the captured image; and 7, anti-vibration table surface for stability of image.

An experimental setup for contact angle measurement is shown in Figure 10. The setup consists of two double convex optical lenses (focal length 600 mm) placed between a highspeed camera (Guppy F-503 B&W CMOS, Allied Vision Technologies GmbH, Stadtroda, Germany with 7.5 frames/s and 2592 × 1944 resolution) connected with a computer system that has an image capture and analysis interface. The camera positioning and specimen with droplet can be set parallel to each other for a clear view of the droplet. All parts were arranged on the table, isolated from external vibrations and disturbances in order to maintain the stability and quality of the images for accurate measurement.

**Figure 11:**
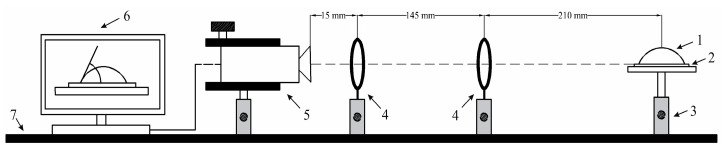
Illustration of the critical distance between parts for angle measurements: 1, droplet; 2, analyzed AAO specimen; 3, adjustable stand for specimen; 4, double convex lenses; 5, Guppy F-503 high speed B&W CMOS camera; 6, computer system for analysis of the captured image; and 7, anti-vibration table surface for stability of the image.

For accurate image vision and stability, the acritical distance between camera, convex lenses, and the specimen with a droplet, is very important (Figure 11).

The experiment was performed in an ambient light source in a dark laboratory room. The light settings were arranged so that the liquid drop would appear black, which was necessary for measurement accuracy as well as for image analysis. Additionally, the experiment was arranged to avoid any reflection of light that could spoil the measurement. Precautions were also taken to prevent the drops being polluted by airborne impurities such as dust and particles.

**Figure 12:**
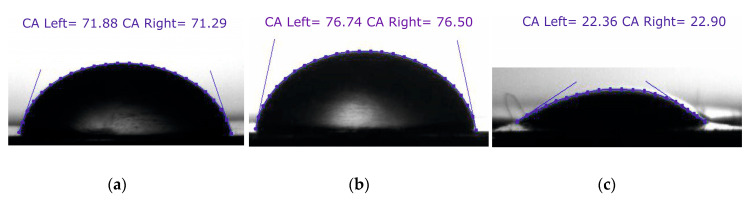
Polynomial fit of the droplet profile through knots starting from the left lower end to the right lower end with the contact angle; (**a**) distilled water; (**b**) glycerin; (**c**) spirit.

First, the height of the specimen holder was adjusted according to the parallel camera vision for an accurate position of the droplet image. Then, a 20 μL droplet of distilled water was diffused using pipet on the analyzed nanoporous AAO membrane surface from a height of 15 mm. Immediately after, the stabilized image of the diffused droplet was captured by the camera interface. For image processing, ImageJ software (University of Wisconsin, WI, USA) with DropSnake plugins freely available and provided by Wayne Rasband (retired from NIH) was used. This software provides an easy interface for image processing and provides accurate quality results. This method obtains contact angle by using a polynomial fit for obtaining the curve of the droplet profile. The method is as follows: start by putting 7 knots from the left lower end to the right lower end along the profile of the droplet, which should cover the inside of all knots, as shown in Figure 12. Then, click twice on the image that will demonstrate the estimated values of the left-hand and right-hand side angles and click on the green play button to redefine the drop profile and accept it.

Three different liquids (distilled water, glycerin, and spirit) were tested on a fabricated nanoporous AAO membrane (Figure 12). The quantity of diffused droplet was kept constant for each measurement and experiments were performed several times.

**Figure 13:**
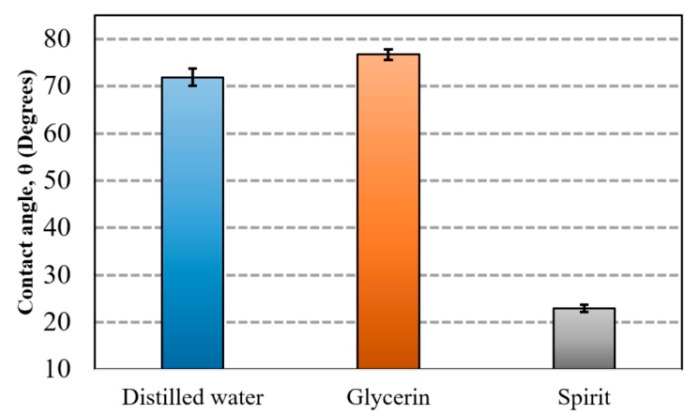
Mean contact angle on the surface of the nanoporous AAO membrane for distilled water, glycerin, and spirit.

A graphical representation of measured contact angle (*θ*) for different fluid droplets on a nanoporous AAO membrane is presented in Figure 13. The measured value of the contact angle formed by water droplet was 71.88° with a measurement error of ±1.25°. The maximum contact angle was observed for glycerin at 76.74° with a measurement error of ±1.85°. The lowest contact angle was observed for spirit at 22.93° with a measurement error of ±0.4°. According to the literature [53,54], a material is considered to be hydrophobic if the water contact angle is ≥90°; if it is ≤90° , it is considered to be hydrophilic (the surface has more wettability or spreading). Thus, our experimental results state that the surface of the fabricated nanoporous AAO membrane falls within the range of hydrophilic material with water because its contact angle is less than 90°.

A calculation of the confidence level interval for the error in measurement was made using Equation (3). The value of *Z* was taken from the normal distribution 1.96, because the measured values lie within the range of the standard deviation of the mean. Also, the standard error of the mean value was calculated using Equation (4).

(3) $$Confidance\;interval=\bar{x}\pm Z\frac{s}{\sqrt{n}}$$

where $\bar{x}$ = average of measurement, *Z* = 1.960 constant for accuracy level 95%, *s* = standard deviation, and *n* = number of measurements.

(4) $$Standard\;error\;of\;mean\;(\sigma_{m})=\frac{s}{\sqrt{n}}$$

where *s* = standard deviation and *n* = number of repetition of measurements (i.e., rate of repetition was 15).

**Figure 14:**
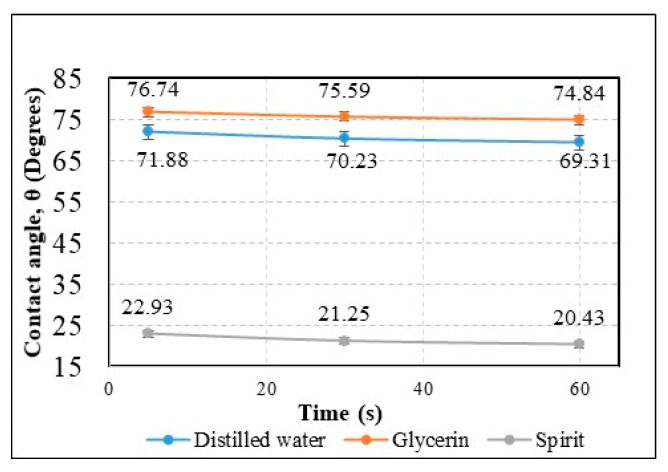
Time dependence of the contact angle for different fluid droplets on the AAO membrane.

Additionally, the contact angle was measured after 5 s, 30 s, and 60 s (Figure 14) for different liquids (distilled water, glycerin, and spirit) on the surface of the AAO membrane to observe the spreading of a droplet.

A gradual decrement was observed for the water contact angle (θ) 71.88°, 70.23°, and 69.31° at 5 s, 30 s, and 60 s, respectively. Similarly, a gradual decrement was observed in the contact angle for the glycerin and spirit droplets after 5 s, 30 s, and 60 s, as shown in Figure 14. After 60 s, the droplets of all fluids were found to be stable, i.e., there was no change in the contact angle.

## 4. Numerical Simulation and Experiment Analysis of Nanoporous AAO Membrane Actuation for Nanofiltration

A novel nanoporous AAO membrane was designed with the intention of using it for filtration and separation in microfluidic devices. To confirm its suitability, a numerical simulation was conducted using surface acoustic waves (SAWs) in the form of standing acoustic waves for acoustophoretic particle separation and manipulation in a micro scale dimension [44,45].

### 4.1. Numerical Simulation for Nanoporous AAO Membrane Actuation

**Figure 15:**
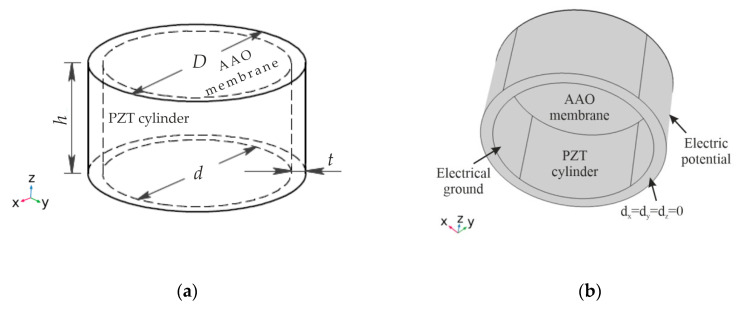
Simulation model (**a**) and boundary condition (**b**) of the finite element model of the AAO membrane.

The behavior of the fabricated nanoporous AAO membrane was investigated numerically using COMSOL Multiphysics 5.4 software. A three-dimensional numerical model (Figure 15) was constructed to elucidate the change in the displacement of the nanoporous AAO membrane surface, when excited with SAW in the presence of amplitude modulation. The simulation model (Figure 15a) consisted of a piezoelectric cylinder (PZT-5H, inner diameter *d* = 30 mm, thickness *t* = 2 mm, and height *h* = 15 mm), used as an actuating member, and the fabricated nanoporous AAO membrane (diameter *D* = 30 mm and thickness 0.16 mm) on top. The boundary conditions of the analyzed model are given in Figure 15b, and the properties of the piezoelectric material are presented in Table 3.

**Figure 16:**
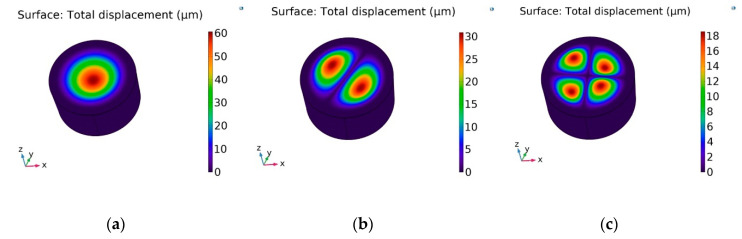
Surface displacement field of the membrane with an operating frequency of: (**a**) 3.50 kHz at 0.44 V (**b**), 4.94 kHz at 1 V (**c**), and 7.89 kHz 2.5 V.

The results of the dynamic response of the membrane, actuated by using PZT-5H, are presented in Figure 16. A deformation of the membrane, generated with an electrical excitation of 0.43 V at a frequency of 3.5 kHz (the first natural frequency mode), is shown as the total displacement field (Figure 16a). It can be observed that the geometry of membrane has displacement, mainly concentrated in the center, i.e., the membrane oscillated in half wave mode. Meanwhile, in the second mode, the natural frequency of system was achieved with an electrical excitation of 1 V at a frequency 4.94 kHz, with two halves of the membrane on the surface with 180 shifts in phase (Figure 16b). The membrane surface with four quadrants was achieved at 7.89 kHz frequency (Figure 16c). Therefore, it is possible to state that the geometry of the nanopores can be controlled by employing vibration. The acceleration of driving frequency leads to an increase in the concentration of the particle near the center of the pores and reduces friction, which may damage the cell membrane.

### 4.2. Experimental Analysis of Nanoporous AAO Membrane

**Figure 17:**
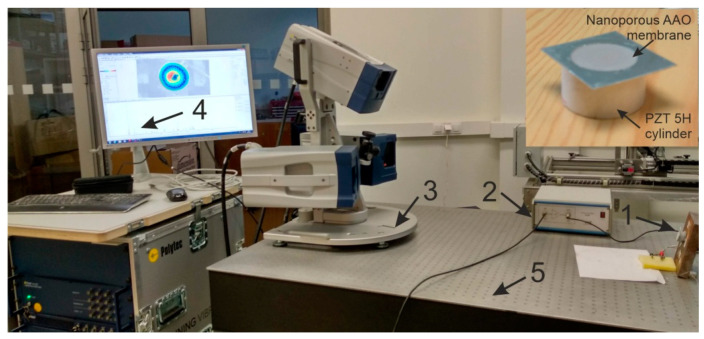
Experimental setup (3D scanning vibrometer PSV-500-3D-HV) for the actuation of the membrane: 1, nanoporous AAO membrane fixed on PZT 5H cylinder as shown right top corner; 2, voltage amplifier; 3, 3D scanning vibrometer (PSV-500-3D-HV, Polytec GmbH, Waldbronn, Germany); 4, computer system with software; and 5, anti-vibration table.

**Figure 18:**
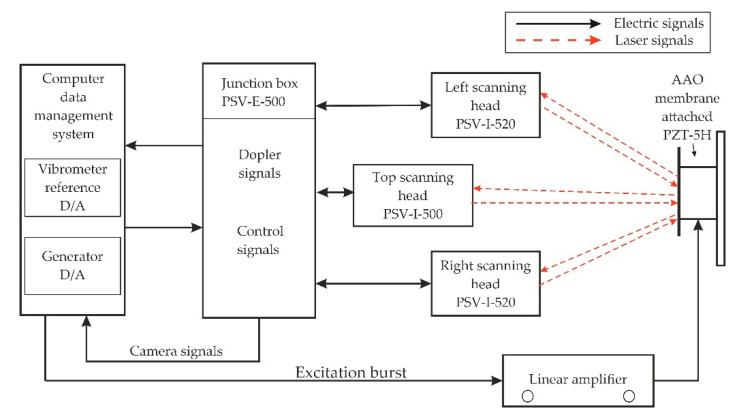
Illustration of the experimental setup for the actuation of the membrane using a Polytech PSV-500-3D-HV laser vibrometer.

The goal of this experiment was to validate the operating principle of the actuator and to verify the vibration modes and operating (resonance) frequencies. Frequency response analysis was done using a non-destructive testing method, 3D scanning vibrometer (PSV-500-3D-HV, Polytech, Germany). The experimental setup (Figure 17) consisted of a piezoelectric actuator, a voltage amplifier, a 3D vibrometer PSV-500-3D-HV, a computer interface with analysis software, and anti-vibrating table (surface isolated from any external forces). A prototype model of the piezoelectric actuator (inset Figure 18) as a vibro-active-nano filter consisted of PZT 5H cylinder (diameter 30 mm, thickness 2 mm, and height 15 mm) with a membrane (diameter 30 and thickness 2 mm) placed on the top of the cylinder.

The experiment was performed under stable conditions, i.e., no external vibrations acting on the membrane. The PZT actuator was fixed on the steel plate with the help of glued tape and placed on the anti-vibration table. The PZT 5H cylinder was connected to the voltage amplifier by an electrode for voltage amplification. Using a computer system with integrated with software, points were set with the Polytech laser at the periphery of the aluminum oxide membrane covering the measurement geometry (shown in Figure 17, computer screen). Vibrations in the actuator were generated by electrical signal 5 V. A schematic of the experimental setup with the 3D scanning vibrometer is shown in Figure 18.

The PSV-500-3D-HV laser vibrometer (Polytec GmbH, Waldbronn, Germany) comprises of three laser sensor heads. The examining head (PSV-I-500) with high accuracy is called the top head, which contains the full HD camera (20× zoom) for perception, arrangements, and video triangulation, as well as the geometry filter unit (PSVG-500, Polytec GmbH, Waldbronn, Germany). There are two filtering heads (PSV-I-520, Polytec GmbH, Waldbronn, Germany), which do not contain the camcorder and geometry scan unit and are indicated as left/right scanning heads. The front-end (PSV-F-500-V, Polytec GmbH, Waldbronn, Germany) unit has three computerized broadband decoders and a sign generator. The high frequency doppler signal originating from the filtering head is transmitted to the decoder and the velocity data is extricated. The junction box (PSV-E-530 Polytec GmbH, Waldbronn, Germany) works as an interface between the scanning heads and the front-end unit. The estimation information is transferred to the computer system utilizing an ethernet interface. The three laser heads must be adjusted to one another and to the measuring object (AAO membrane) before making the measurements.

**Figure 19:**
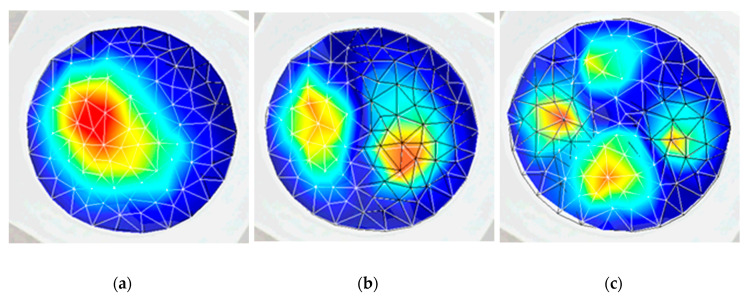
Experimental result (3D scanning vibrometer PSV-500-3D-HV) response of the actuator on the surface of the membrane at different frequencies: (**a**) 3.62 kHz (first mode of vibration); (**b**) 5.94 kHz (second mode of vibration); and (**c**) 7.89 kHz (third mode of vibration).

The result of the response of the PZT actuator is presented in Figure 19. A deformation of the membrane was electrically generated at 3.62 kHz (the first mode of vibration) and is shown in Figure 19a. It can be observed that the shape of the deformation is similar to the shape obtained during the numerical simulation at 3.5 kHz frequency. The second mode of vibration (Figure 19b) was observed at 5.34 kHz frequency, confirming simulation results with 4.94 kHz. The third mode of vibration (Figure 19c) was obtained at 7.45 kHz, while, in the numerical simulation, the third mode was observed at 7.89 kHz. Thus, difference between simulation and experimental frequencies for the first, second, and third modes are 3.3%, 7.7%, and 5.7% respectively. The error mainly occurs due to the inaccuracy of material properties, neglecting the glue layer between the PZT cylinder and the membrane.

**Figure 20:**
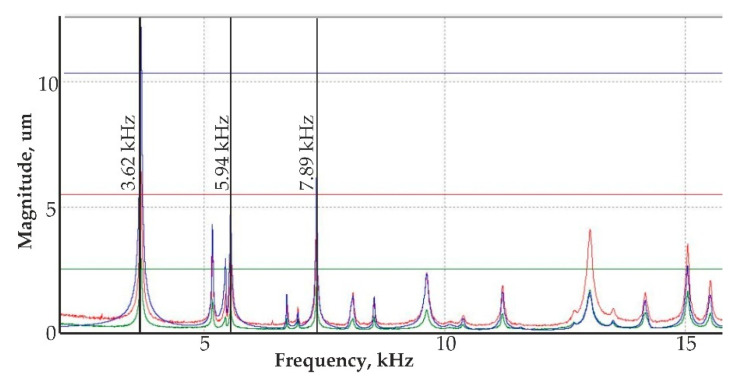
Resonance mode of the actuator from 3D vibrometer frequency response characteristics: a resonance mode of the actuator at 3.62 kHz, 5.94 kHz, and 7.89 kHz with different colors showing displacement directions: x – green, y – red and z – blue.

Analyzing the vibration model of the PZT actuator with the 3D vibrometer, three different shapes of the model were achieved. A resonance mode of the actuator at different magnitudes is presented in Figure 20. The displacement magnitude in y-z plane of the nanomembrane was determined at different frequency ranges.

A non-contact holographic measurement system known as PRISM, with the PZT-5H actuator was used to validate the simulation results with the experimental results of the actuated membrane. A holographic approach can be employed for a visual illustration of the dynamic processes, occurring in the waveguide of the optical scanner. This is the most effective method for studying these dynamic processes [55].

**Figure 21:**
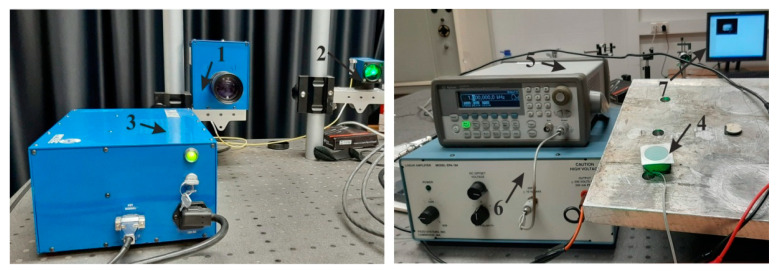
Experimental setup of the holographic interferometry (PRISM) of the membrane: 1, video head; 2, illumination head of the object; 3, control block; 4, aluminum oxide membrane mounted on Piezoelectric cylinder; 5, frequency generator; 6, voltage generator; and 7, interference fringe (computer screen).

The PRISM system for surface deformation of membrane, actuated by PZT-5H actuator, is shown in Figure 21. It consists of the equipment for the deformation (frequency generator and voltage generator) and vibration measurements (holography), as well as a computer system with software.

**Figure 22:**
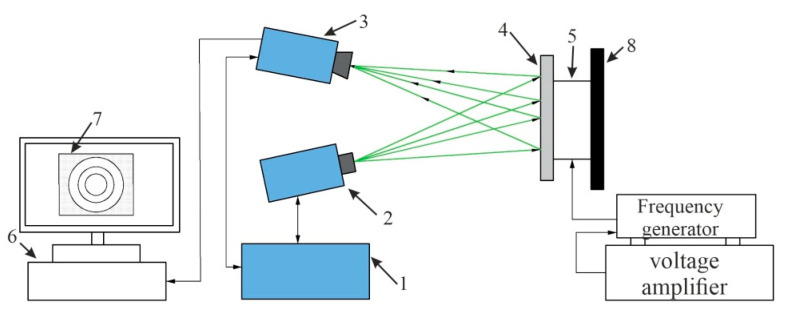
Experimental setup of the PRISM system for the membrane: 1, control block; 2, illumination head with green laser; 3, camera; 4, membrane attached on PZT actuator; 5, PZT-5H actuator; 6, computer fringe; 7, computer screen (image illusion); 8, isolated surface for fixing object; and frequency and voltage amplifier for the actuation of the membrane.

An illustration of the experimental set up of the holographic interferometry PRISM system for the actuation of the membrane is shown in Figure 22. A two-beam speckle pattern interferometer (Figure 22) with a green laser (wavelength 532 nm, power 20 mV) was used. In this set-up, there are two beams directed on the actuating membrane: an object beam, which is directed onto the membrane, and a reference beam, directly captured in the camera. A laser light is then scattered from the object and accumulated by the camera lens, picturing the object onto the CCD (charge-coupled device) camera sensors. An image of the object (membrane) is then transferred from the camera to the computer system and analyzed with a program PRISM DAQ (Hy-Tech Forming Systems (USA), Phoenix, AZ, USA). The adynamic response on the surface of membrane at difference frequencies is observed in the computer screen.

**Figure 23:**
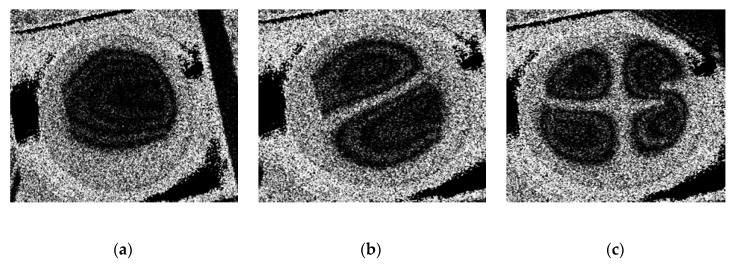
Experimental (holographic interferometry PRISM) response of the actuator on the surface of the membrane at different frequencies: (**a**) 3.8 kHz (first mode of vibration) at 0.43 V; (**b**) 5.18 kHz (second mode of vibration) at 0.44 V; and (**c**) 8.06 kHz (third mode of vibration) at 2.12 V.

The obtained modes of the deformed surface of the membrane at different frequencies are given in Figure 23. The first mode (Figure 23a) was obtained at 3.8 kHz frequency with 0.43 V; a similar deformation was retrieved during the simulation at a frequency of 3.50 kHz (0.44 V). An error of 8.2% was observed between the numerical and experimental results. Meanwhile, the second mode (Figure 23b) of vibration was at 5.18 kHz (0.44 V) and 4.94 kHz (1 V), with an error of 4.7% for the experimental and numerical results, respectively. The third mode (Figure 23c) of vibration for the membrane was achieved at 8.06 kHz (2.12 V) and 7.89 kHz (2 V) with a 2.1% error in the experimental and numerical simulations, respectively.

Thus, the numerical and experimental study for the actuation of the membrane, using a PZT-5H actuator, shows the superior distribution of electromechanically standing waves on the surface of membrane at different frequencies. Further, a numerical simulation of bio-particle separation and manipulation was done applying the acoustophoresis method.

**Figure 24:**
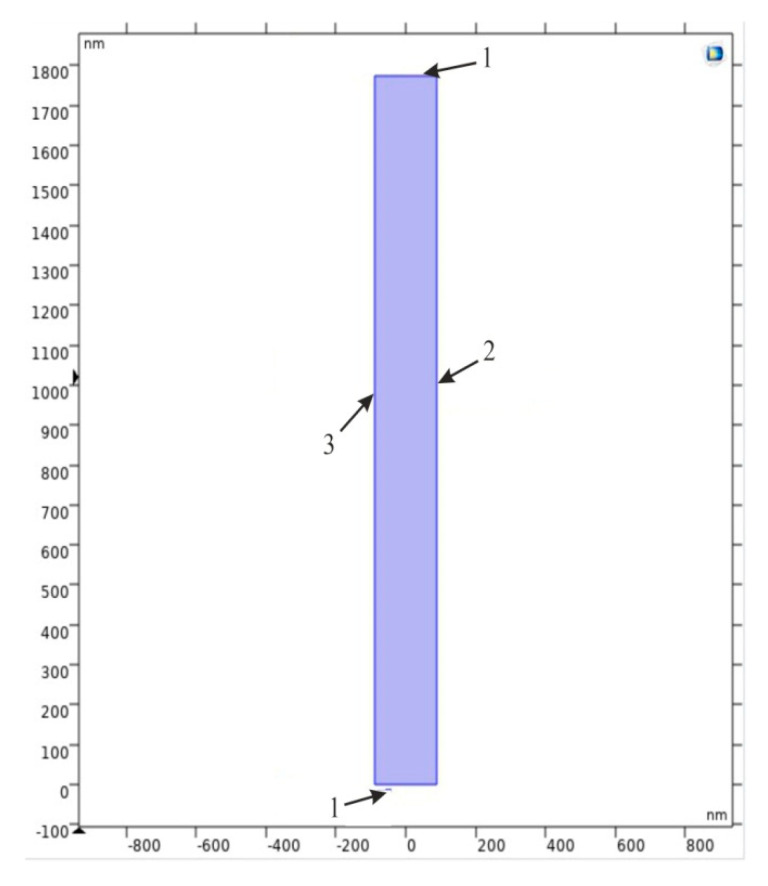
Simulation 2D model of nanocavity: 1 - plane wave radiation; 2 - normal acceleration; and 3 - sound hard boundary.

A formed nanoporous AAO membrane was proposed for the ultrafiltration of the nanoparticles in the fluid of the micro-hydro-mechanical systems. Therefore, a simulation was performed to determine the acoustic pressure distribution, particle motion, and operating frequencies for the nanotube, distributed in the AAO membrane. Using the acoustophoresis method, a symmetrically distributed nanocavity of hexagonal tube shape with a rectangular cross-section (2D model) (shown in Figure 24) was selected for the simulation of the bioparticle. For this purpose, COMSOL Multiphysics 5.4 was used. The main parameters for the simulated model are presented in Table 4.

**Figure 25:**
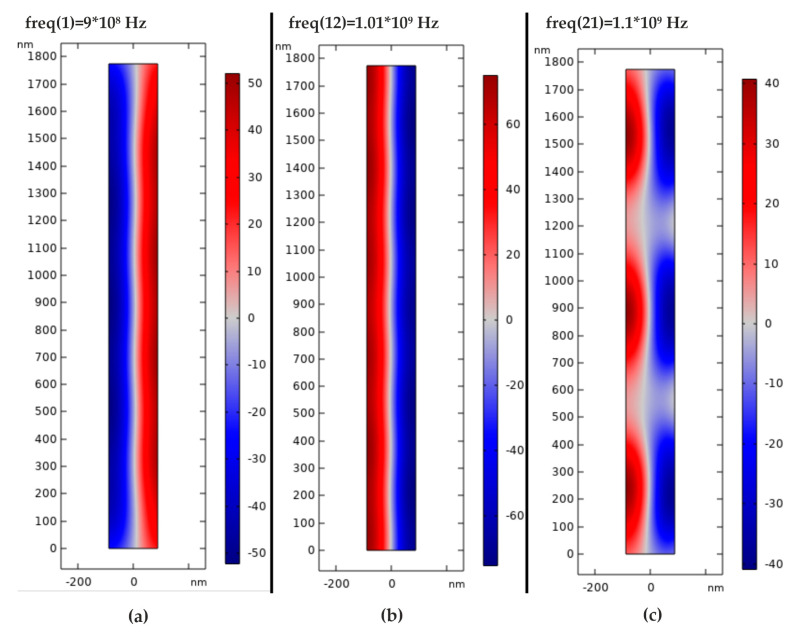
Acoustic pressure distribution in a nanochannel excited at: (**a**) 900 MHz; (**b**) 1001 MHz; and (**c**) 1100 MHz.

The numerical model was divided into finite elements with a maximum size of λ_0_/2.5 and a minimum element size of 5.32 × 10^−6^ nm. This simulation model had lamination, which made the simulation results similar to those of a real model. The calculations of the model were divided into two steps. The first study was devoted to Eigen frequency calculations, in which nanoparticles go through the microchannel without touching wall of the channel, i.e., nanoparticles pass though the center of channel. Therefore, calculations were made to find the frequency at which the particles focus in the center of the channel in the range from 900 MHz to 1100 MHz by 10 MHz step. The obtained result of the distribution of acoustic pressure inside the channel are presented in Figure 25.

**Figure 26:**
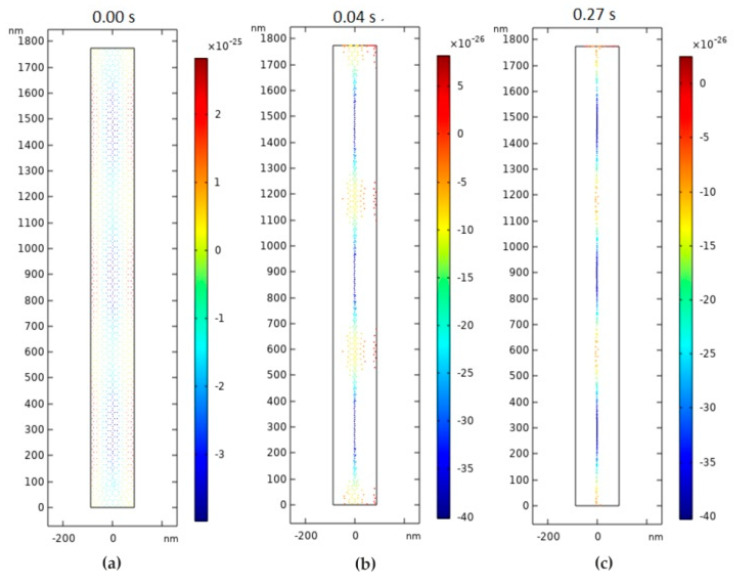
Nanoparticle positioning inside nanochannel: (**a**) 0 s; (**b**) 0.04 s; and (**c**) 0.27 s.

A form of the transverse oscillation at 900 MHz frequency provides the lowest acoustic pressure in the center of channel (Figure 25a), which means that the nanoparticles are concentrated in the center of nanotube. The nanoparticle responds to primary acoustic radiation force when dispersed in an ultrasound standing wave by moving to specific locations along the wave. These results were used for further analysis. An increment in the frequency results in the disorder of the acoustic pressure inside the nanochannel, leading to an increase of friction between the particle and nanochannel wall, while passing through it (Figure 25b,c). The difference in pressure affects the movement of the particles and orients them in the center. A second study was performed to determine how long it takes for a nanoparticle to focus on the center of a nanochannel. For this purpose, the analysis time was chosen from 0 s to 0.3 s by 10^−4^ s step. The obtained results (Figure 26c) showed that the nanoparticles with a diameter of 23.65 nm and a density of 500 kg/m^3^ (such as the nanoparticles of dust, sawdust, and carbon) focused in the center of this nanochannel in just 0.27 s.

**Figure 27:**
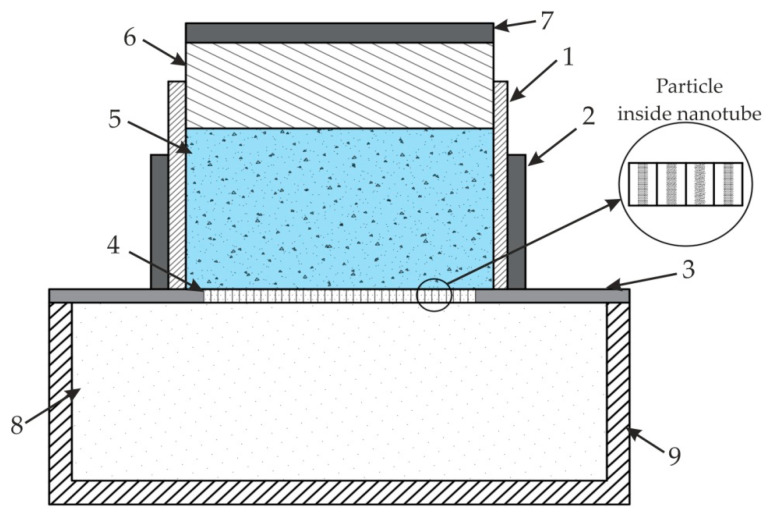
A model for nanoparticle separation: 1, container with nanoparticle and fluid; 2, PZT actuator; 3, AAO attached with PZT actuator; 4, nanotubes AAO; 5, fluid with nanoparticles; 6, plunger for pressure; 7, PZT disc; 8, separated nanoparticles; 9, nanoparticle collector.

The simulation results of the nanochannel showed that transverse oscillation appears in the channel and particles are concentrated in the center of it at a minimum pressure. It allows particles to be aligned, separated, or sorted inside the nanochannel due to the lower acoustic pressure that develops in the center of nanotube. Thus, the results confirmed that a fabricated nanoporous AAO membrane could be used for bioparticle manipulation using the acoustophoresis method. An example of a system for nanoparticle separation from fluid is shown in Figure 27.

A proposed nanoparticle separation model may be used for particle ultrafiltration. The system consists of a container with nanoparticles, fixed in a piezo-electric ceramic cylinder (actuator) with a nanoporous membrane, consisting of nanotubes. The assembled plunger with a PZT disc on top of it is placed for acoustic pressure generation. A whole assembly is then attached to a nanoparticle collector at the bottom. Ultra-high frequency vibration is used to focus the nanoparticle on the center of a nanocavity to pass through nanotubes, and to keep the filter from clogging. Moreover, standing acoustic waves acting on the surface of membrane (as shown in Section 4) help to manipulate the nanoparticle mixed with fluid (i.e., it could be shorted, separated, or filtered from the liquid due to acoustophoresis pressure) passing through the nanotube. Thus, surface acoustic waves with ultra-high frequency will prevent bioparticles from developing friction and damaging the nanochannel wall.

## 5. Conclusions

In this research paper, the development of a nanoporous AAO membrane for the separation of particle in micro hydro mechanical systems, using the acoustophoresis method, is presented.

1. The nanoporous AAO membrane was successfully fabricated using two-step anodization method (MA and HA) in 0.3 M oxalic acid at 60 V with a hexagonal structure having a thickness of 120 µm with 70 nm pore diameter, 110 nm interpore distance, 36.72% porosity, and 4.771 cm^−1^ × 10^9^ pore density.
2. Fourier-transform infrared spectroscopy (FTIR) results confirmed that nanoporous structures were obtained in AAO membrane. SEM based EDS analysis confirmed that the nanoporous honeycomb hexagonal structure was formed on the membrane and microanalysis using EDS spectrum showed the dominance of aluminum (36.7%) and oxygen (59.5%) in the fabricated AAO membrane. Other peaks showed the smaller quantity of carbon (2.8%), sulfur (0.55%), and phosphorus (0.45%).
3. The hydrophobic/hydrophilic properties were analyzed by measuring the water contact angle on the surface of the AAO. The contact angle was formed with the water droplet at 71.88° ± 1.25°. This indicated that the formed membrane is a hydrophilic material with water (since the water contact angle was <90). The contact angle measured for glycerin was 76.74° ± 1.85° and for spirit, it was 22.93° ± 0.4°, i.e., less than 90°, meaning that this membrane has a hydrophilic surface. Additionally, a contact angle time dependence study was conducted after 30 s and 60 s, and a gradual decrement of approximately 1° was observed in both cases. This indicated the seeping of the liquid droplet into the AAO membrane pores.
4. A simulation of acoustic wave distribution was done with a COMSOL Multiphysics 5.4. The different forms of wave formation on the surface of the nanomembrane were observed: a first mode at 3.50 kHz, a second mode 4.94 kHz, and a third mode 7.89 kHz. This phenomenon enables the internal geometry to be controlled using standing surface acoustic waves of the nanoporous membrane and allows the nanoparticles to be concentrated in the center of the nanotubes.
5. Theoretical results of the actuated nanomembrane were verified with experimental results. Similar vibration modes using a 3D vibrometer and holographic interferometry PRISM system were obtained at the following frequencies: a first mode at 3.62 kHz and 3.8 kHz, a second mode at 5.94 kHz and 5.18 kHz, and a third mode at 7.89 kHz and 8.06 kHz, respectively, for the simulation and experimental results. An error occurred in the simulation and experimental results because of the glued layer between the PZT 5H cylinder and nanoporous membrane.
6. The simulation results for single nanotube for acoustic pressure distribution showed that the lowest pressure is in the center of the channel at 900 MHz. It allows the bioparticle to pass through a nano tube/channel without friction with the walls because of the lower acoustic pressure acting in the center of nanotube. Moreover, it takes 0.27 s to focus the nanoparticle into the center of channel, reducing the possibility of damage to the cell membrane.
7. A model for the nanoparticle filtration, using standing surface acoustic waves generated by a PZT 5H cylinder (actuator), was proposed. This model could be implemented as a vibro-active nano filter in a biomedical micro hydraulic mechanical system.

## Figures and Tables

**Table 1:** Characteristic parameters of a fabricated nanoporous aluminum oxide membrane.

Anodizing Potential (V)	Mean Pore Diameter, *D*_p_ (nm)	Interpore Distance, *D*_c_ (nm)	Porosity (%)	Pore Density (cm^−1^ × 10^9^)
60	70 ± 20	110 ± 10	36.72	4.771

**Table 2:** Chemical composition of the nanoporous AAO membrane.

Chemical Element	Normalized Concentration in Weight Percentage (norm.wt., %)	Normalized Concentration in Atomic Percentage (norm. at., %)
Carbon	2.8	4.5
Oxygen	59.5	69.55
Aluminium	36.7	25.39
Sulfur	0.55	0.29
Phosphorus	0.45	0.27

**Table 3:** PZT 5H material properties.

Property (Symbol), Unit	Value
Coupling coefficient (k_33_)	0.75
Displacement coefficient (d_33_), m/V	650 × 10^−12^
Voltage coefficient (g_33_), V m/N	19 × 10^−3^
Density, kg/m^3^	7800
Young’s modulus, N/m^2^	5.5 × 10^10^
Poisson’s Ratio	0.34
Mechanical Q factor	32

**Table 4:** 2D model simulation parameters.

Parameter	Values
Driving frequency, Hz	5.8 × 10^9^
Speed of sound, m/s	343
Wavelength, m	5.9138 × 10^−8^
Transducer diameter, m	1.1828 × 10^−7^
Reflector diameter, m	1.7741 × 10^−7^
Height, m	1.7741 × 10^−6^
Viscous boundary layer thickness, m	2.8887 × 10^−8^
Particle diameter, m	2.3655 × 10^−8^
Particle density, kg/m^3^	500
Normal acceleration of transducer, m/s^2^	1.5 × 10^−8^
